# Ten years of integrated care for the older in France

**DOI:** 10.5334/ijic.668

**Published:** 2011-12-14

**Authors:** Dominique Somme, Matthieu de Stampa

**Affiliations:** Paris Public Hospital Service, Georges Pompidou European Hospital, Department of Geriatrics, 20-40 rue Leblanc, 75015 Paris, France; Health Environment and Aging, National Institute of Health and Medical Research, EA 2506, Université de Versailles Saint-Quentin, Paris, France; Paris Public Hospital Service, Sainte Périne Hospital, Department of Geriatrics, 11 rue Chardon Lagache, 75781 Paris Cedex 16, France; Health Environment and Aging, National Institute of Health and Medical Research, EA 2506, Université de Versailles Saint-Quentin, Paris, France

**Keywords:** France, health and social integration, gerontology

## Abstract

**Background:**

This paper analyzes progress made toward the integration of the French health care system for the older and chronically ill population.

**Policies:**

Over the last 10 years, the French health care system has been principally influenced by two competing linkage models that failed to integrate social and health care services: local information and coordination centers, governed by the social field, and the gerontological health networks governed by the health field. In response to this fragmentation, Homes for the Integration and Autonomy for Alzheimer patients (MAIAs) is currently being implemented at experimental sites in the French national Alzheimer plan, using an evidence-based model of integrated care. In addition, the state’s reforms recently created regional health agencies (ARSs) by merging seven strategic institutions to manage the overall delivery of care.

**Conclusion:**

The French health care system is moving from a linkage-based model to a more integrated care system. We draw some early lessons from these changes, including the importance of national leadership and governance and a change management strategy that uses both top-down and bottom-up approaches to implement these reforms.

## Introduction

As in many developed countries, France has a growing number of older people with chronic conditions, and this is challenging the re-organization of long-term care [1]. This demographic shift has revealed the fragmented nature of the French healthcare system, which is mainly focused on acute care. We have identified fragmentation between health and social services; between institutional (hospital and long-term care) and community-based care services; between private, non-profit and public services; and between the various payment systems (public, insurances, fee-for-services) [2]. The fragmentation is noticeable at all levels of responsibility, even at the national level. No single institution is able to determine gerontology policy, which may explain why the French system is so difficult to reform [3].

This paper provides an update on recent reforms of integrated care in France for the older and chronically ill population. We have used the definition of integration proposed by Kodner and Kyriacou [4]: “We consider integrated care to be a discrete set of techniques and organizational models designed to create connectivity, alignment and collaboration within and between the cure and care sectors at the funding, administrative and/or provider levels.” Our focus is on how reforms have been implemented in a very fragmented healthcare system and what we have learned for the future.

## Context

France has over 65 million inhabitants, 21.3% of whom are 60 years of age and over, and there is great disparity in the availability of services [5]. The country is divided in 26 regions, 100 departments and more than 36,000 municipalities. The healthcare system is decentralized, with a subnational level of governance (by region and department) and few links between social services and health care.

At the national level, two ministries (the ministry of health and the ministry of solidarity) and two insurance systems (health and retirement pension) are responsible for elderly people with chronic conditions.

Until 2010, the health aspects of long-term care were managed by decentralized administrations (at the regional and department levels) that remained under the government’s supervision (DRASS^1^ and DDASS^2^). The regional institutions for hospitals (ARH^3^) organized the development of the public and private structures on their service areas. The regional health insurance providers (URCAM^4^ and CRAM^5^) are the main strategic actors funding health services (primary care with a general practitioner and institutional care). The new reform ‘Hospital, Patients, Health Territories,’ adopted on June 24, 2010, has created a unique agency at the regional level (ARS^6^) that unites all these institutions. The role of the 26 ARSs is to pilot and govern the overall delivery of care in close collaboration with the social services sector.

The social aspects of long-term care are managed mainly by the general councils at the department level following the decentralization policy. The general councils fund social allocations for high chronic impairments (APA^7^) from a national autonomy fund. They also regulate care services (agreements and price setting) within the department. In addition, retirement insurance providers and municipalities can decide to fund and organize the implementation of home assistance services for elderly people with low levels of impairment. Since 2006, some aspects of the regulation of care are under the control of a general practitioner (GP) who, for certain aspects of care, plays a ‘gatekeeper’ role. Patients who use care services without being referred by their GP are not reimbursed as well as those referred by their GP (this is called the ‘coordinated care pathway’). The reform seems to ‘institutionalize’ previous professional and personal attitudes without effecting profound change [6] and without providing any solutions for the identified fragmentation of services. We should also mention that the French system is open to the private sector. For a fuller analysis that takes into account more of the historical factors, we refer readers to the work of other authors [3, 7, 8].

## Recent reforms to integrate care

### The ‘CLICs’: local information and coordination centers

The first major policy of the decade was implemented during the 1999 United Nations International Year of the Elderly. According to the preamble of the circular establishing local information and coordination centers (CLICs), they were introduced “*to rethink our provision (home support policies) and to strive to make it more coherent through the creation of a gerontological coordination network organizing the linkages in national provision from local level upwards”* [9]. In addition to strengthening links between providers, these schemes met the clinical objectives of integration as defined by Kodner and Kyriacou [4]: “*The local information and coordination centers (CLICs) have a multidisciplinary vocation encompassing all aspects of the daily life of the elderly in terms of care, personal support and the quality and user-friendliness of the built environment (environment/habitat) but also social, cultural and civic life”* [9]. Their rollout has been gradual, with an experimental phase involving 25 sites, then expanding at the rate of 200 sites per year toward a national target of 1000 centers. The CLICs were intended to reinforce links between professionals at the clinical and service levels by providing a single entry point function and by creating a framework of joint responsibility between the main actors (government and departmental authorities) [10]. However, when the law was passed on August 13, 2004, the state completely withdrew from its responsibilities and the funding of these centers by instituting a fiscal transfer to the general councils [11]. This absence of the state has had important implications, since the entire health system is still under its direct jurisdiction but social services are largely under the supervision of departmental authorities. Five years later, the development of CLICs has been assessed as unsatisfactory due to an unevenness of implementation across the national territory with the number of centers per department ranging from 1 to 24 [12]. Moreover, CLICs failed to build links between the social service sector and the health sector due to a lack of partnerships at local level [13].

### Gerontological health networks

The National Pension Insurance Fund published a circular in 1993 devoted to the promotion of networks. The networks were included in by-laws to reform the health system in 1996 [14], then entrusted to state health insurance joint funding in 1999 [15]. The 2002 law [16] described their mission thus: *“Health networks are designed to promote access to care, coordination, continuity or the interdisciplinary work of health responsibilities, especially those specific to certain sectors of the population, diseases or health activities. (...) They comprise private health professionals, occupational doctors, health facilities, health cooperation groups, health centers, social institutions or medico-social and health organizations and users’ representatives*.” The very wording of the law is consistent with the definition of integration proposed by Kodner and Kyriacou [4]. The gerontological health networks are mainly focused on the clinical level in order to provide a better fit between services and the needs of elderly people living in the community. They implement multidisciplinary teams, including nurses and geriatricians, for older people with complex care needs. But the collaboration with other clinical partners is not formalized, and there is a lack of general practitioners’ participation and strong competition between care providers without limitations placed on their areas of competency. The networks’ funding comes from government and health insurance. They do not create linkages with social authorities at the department level, and they are considered costly [17].

### Homes for the Integration and Autonomy for Alzheimer patients (MAIA^8^)

The 2008–2012 National Alzheimer's Plan attempts to introduce case managers to cater to the needs of the elderly population with complex health situations [18]. It uses an intensive model of case management [19] implemented on a trial basis in an integrated scheme. The Plan is responsible for care and services at the local level (Homes for the Integration and Autonomy of People Suffering from Alzheimer's or Associated Disorders, abbreviated in French as MAIA). In recent years, the French system has studied the potential of case management, often taking disease management as a starting point, as reflected in a report by the General Inspectorate of Social Affairs [20]. Some recent experiments in France at the local level [21] or at multiple sites [22] have tested the integration paradigm. The preliminary results have been promising, prompting decision makers to consider implementing this organizational model at a larger scale.

The MAIA program is based on a coordination-type model similar to the PRISMA model developed in Québec. It has six components (coordination between stakeholders, a case management process, a single entry point, a standardized multidimensional assessment, an individualized service plan, and a shared information system) [23, 24]. The model attempts to implement co-responsible partnership as the main basis for the development of shared tools, clinical practices, care processes and information systems and even shared governance.

The MAIA program relies explicitly on integration (the first time the term has been used in public health policy in France). The program uses the following definition of integration: “*Effective coordination of actors and funding bodies designed to simplify the daily lives of sick people, improve the well-being of caregivers and provide the best care and services for all.”* The target population of people with Alzheimer's disease is not used in a restrictive sense, since the plan states:* “These measures are part of general government thinking on improving the management of the dependency state, which will deal with the financial aspects of home care”* [18]. If the partnership was able to develop such an innovative way of managing the supply of services in a given territory, it would be rewarded a ‘quality label’ for all the partners. One central point in the method is that it relies on the supply of all services, not only on willingness to participate. This explains the importance of strong involvement on the part of the strategic authorities.

Between 2009 and 2011, 17 candidate sites were chosen from a sample of 113 files. The candidates were chosen from all across the nation, favoring contrasts between them (administration or not-for-profit organization, large or small territory, urban or rural population, rich or poor region). For each organization chosen, the first task was to engage a ‘local pilot’ to be in charge of analyzing the service area, serving as a link between organizations and implementing the six components, in close collaboration with the national project team. Given this objective, the change management method is of crucial importance. The entire plan is managed by a National Pilot at the highest level of the governance (under the authority of the President of the Republic and beyond the traditional boundaries of the different ministries involved). Under his responsibility a national project team was formed solely to implement the MAIA program.

The initial results from the implementation phase show that the integration concept was well received by all the stakeholders at a majority of the sites. Moreover, they stressed the importance of prolonged national leadership and management, the usefulness of the model comprising six components to structure the change, the importance of having a real decision-making round table at a decentralized strategic level, and the need to eliminate the confusion created by use of the terms ‘Homes’ and ‘Alzheimer.’ Homes are not ‘homes’ but only a label, and they do not cope only with Alzheimer patients but rather with all types of health problems occurring in an older population.

## Discussion

The French system has a long history of attempts to reorganize services for older people, often using a strong top-down approach but with no real governance over time. Previous attempts have also lacked a conceptual model of integrated care and continuous evaluation throughout the entire implementation. They failed to install real coordination, at the clinical level but also at the organization level, between health and social service stakeholders. The MAIA model with two ‘pilots’ (one national and one local) is one way to implement a two-sided approach (top-down and bottom-up). It may reinforce professionals’ participation in service coordination by accounting for local characteristics and fragmentation. The French plan is very explicit about the ‘WHAT’ and the ‘HOW,’ but it calls for a project manager who can find in their partners the most important consequence of the fragmentation they want to address (the ‘WHY’) and can involve all the partners concerned (even those with which the leader organization is not accustomed to working, and, notably, representatives of users and caregivers). So after over 10 years of integration in France, the choice of an appropriate model and how to implement and develop it would appear to be works in progress.

### A model for conducting the integration process

This model relies on six mechanisms and tools that were defined as conditional for making a partnership work in an integrated manner:

Round-table coordination involving all the stakeholders in the medical, psychological, social service, administrative and environmental fields at all levels of responsibility: national, regional, departmental, local, and clinical. This mechanism seeks to overcome the traditional vertical organization of governance. In fact, the objective in implementing this component is to have decision makers concerned not only with the supply of services over which they have authority, but that all decisions makers will address the offer for the entire area served and how well it corresponds to the population’s needs.

The case management process is performed by professionals (case managers) fulfilling a mission to provide long-term follow-up of individuals in complex situations, according to a model of intensive case management [25]. This mechanism acts at the clinical level. It deploys a specific response for people who suffer from the most fragmentation because of their complex needs. To succeed at their work, case managers need to be identified and accepted by all partners. This is why a coordination round table at the tactical and strategic level is a condition to validate the role of the case managers.

An integrated entry point used to standardize access to services: each person with limited autonomy, his or her family, as well as service providers receive the same response, no matter which partner is solicited. The elderly person does not need to learn how the system works, and should be confident that the partner who has received the request will take into account all sources of supply in the service area rather than only his own supply. This is only possible if the round table shares not only standardized information and processes but also information and indicators of dysfunction. This shared information opens the way to shared decisions for a process of continuous quality improvements to access services (in this component, case management is seen as one service like any other).

A standardized multidimensional assessment of all an elderly person’s needs, shared and recognized by the organizations that provide access to services. The challenge is to encompass a traditional ‘compensation way of thinking,’ in which all the difficulties expressed by a client are first seen as difficulties to be compensated for. With a comprehensive assessment, the professional will need to make links between difficulties, analyze them in terms of causes and effects, and, finally, establish a list of problems characterizing the situation. The standardization of the assessment tool allows a more comprehensive assessment (not one based only on memory and expertise) and improves equity in the allocation of resources.

An individualized service plan, developed with the person when he or she is in case management in partnership with the service providers concerned and in consultation with the primary care physician. The service plan is implemented, monitored, and periodically reassessed. Intervention plans are characterized by many professional and non-professional individuals involved in providing assistance; by uncertainty and fluctuating needs and even responses. The tool that case managers must use should help them in their planning.

A shared information system that allows service providers to have access to shared information-sharing procedures relative to the elderly individuals in their care (if they provide their consent). This mechanism is required in order to overcome the usual duplicating of information systems, which explains why professionals, services managers and even policy makers from different parts of a system have different views on a person’s problems, and which problems constitute a priority for action.

The definition of the label and the certification process is currently underway and will be based mainly on the six components.

### An institution to oversee development of service integration

The creation and implementation of ARSs constitute a major reform of the French social services and health care system. The health care and medical/social sectors have an opportunity to be unified in governance, whereas until now they have been compartmentalized by the legal and funding frameworks. The potential of these new organizations to integrate services appears to be real, even if merging institutions does not necessarily lead to integration. This mandatory reorganization of the state and delocalized representation is only defined by law (top-down) and has been placed under the usual governance authority of the Ministry of Health, so there has been no experimental phase. All the executive managers of the ARS were appointed by the government in 2010. Nevertheless, some aspects of the merger and some governance tools have not yet been fully defined, and this has led to some delays in the implementation of ARSs [26].

The ARSs have been designed to carry out the development phase of the MAIA implementation in their service areas in close collaboration with the general councils. They will designate a local pilot at each MAIA site to involve professionals in the process and manage the implementation of the reforms. The pilot will identify local sources of fragmentation to the ARS to improve the re-organization of services and the system’s response to the population’s needs.

Finally, there is an interest in creating a fifth social security risk category in order to provide disposable income to older people in need of long-term care, regardless of their age, so that they can obtain the care and services they need. This follows on work that attempted to integrate public and private funding and possibly remove the age limit, which has historically been defined as retirement age. A broad-based consultation was conducted during the spring of 2011 in view of introducing a profound reform to the funding system. The age limit was not debated as part of this consultation, and the bill that was announced concerned a disability law for the elderly that would involve the public administration and private insurers. More recently, considering the impact of the debt crisis in Europe, the government has canceled this reform, so the next important step for this reform will probably be the upcoming presidential election. This fact illustrates a characteristic of very fragmented health and services system (like the French onethe major importance of political actors on the possibility for the system to change. In such system, during electoral periods, reforms are usually delayed. Moreover, if the reform encompasses multiple political level, the electoral periods are multiple resulting in long delays.

## Conclusion

The MAIA project provides an opportunity to study the implementation and impacts of integrated care at a national scale. At the moment, it is difficult to develop an accurate picture of the provision and integration of care and services for elderly people with chronic conditions in France and regional disparities are always great. The change management used in the MAIA program is innovative in terms of how it reconciles top-down and bottom-up approaches. The concomitance of the ARS implementation seems to be essential to moving the French system toward better service integration since it reduces the complexity of system governance. Nevertheless, the recent history of French reforms briefly reported here and our system analysis underscores the need for strong and continuous national political leadership and national management that can implement a real decentralized strategic round table. These results should help other countries implement their own integrated care policies.

## Footnotes

1 Direction Régional Affaires Sanitaires et Sociales.

2 Direction Départemental des Affaires Sanitaires et Sociales.

3 Agence Régional d'Hospitalisation.

4 Union Régionale des Caisses d'Assurance Maladie.

5 Caisse Régionale d'Assurance maladie.

6 Agence Régionale de Santé.

7 Allocation Personnalisée Autonomie.

8 Maisons Autonomie Intégration Alzheimer.

## References

[1] Robine JM, Michel JP (2004). Looking forward to a general theory on population aging. Journal of Gerontology series. A Biological Sciences and Medical Science.

[2] Henrard J (2002). Le système français d'aide et de soins aux personnes âgées. Santé Société Solidarité.

[3] Trouve H, Couturier Y, Etheridge F, Saint-Jean O, Somme D (2010). The path dependency theory: analytical framework to study institutional integration. The case of France. The International Journal of Integrated Care [serial online].

[4] Kodner DL, Kyriacou CK (2000). Fully integrated care for frail elderly: two American models. The International Journal of Integrated Care [serial online].

[5] Blanpain N, Chardon O (2010). Projection de la population à l’horizon 2060: un tiers de la population âgé de plus de 60 ans. Insee Première.

[6] Dourgnon P, Guillaume S, Naiditch M, Ordonneau C (2007). Les assurés et le médecin traitant: premier bilan après la réforme. Questions d'économie de la Santé.

[7] Couturier Y, Trouve H, Gagnon D, Etheridge F, Carrier S, Somme D (2009). Receptivité d'un modèle québecois d'intégration des services aux personnes âgées en perte d'autonomie en France. Lien social et Politiques.

[8] Ennuyer B (2004). Les malentendus de la dépendance: de l'incapacité au lien social.

[9] Ministère de l'Emploi et de la Solidarité (2000). Circulaire DAS-RV2 n°2000/310 du 6 juin 2000 relative aux Centres locaux d'information et de coordination (CLIC). Expérimentation en 2000 et programmation pluriannuelle 2001–2005.

[10] Frossard M, Genin N, Guisset M, Villez A, Leichsenring K, Alaszewski A (2004). Providing integrated health and social care for older persons in France: an old idea with a great future. Providing integrated health and social services for older persons.

[11] Gouvernement français (2004). Loi n°2004-809 du 13 août 2004 relative aux libertés et responsabilités locales.

[12] Gallez C (2005). Rapport sur la maladie d'Alzheimer et les maladies apparentés.

[13] Colvez A (2010). Génèse et évolution des Clics: expérience des centres locaux d'information et de coordination depuis le bureau d'information gérontologique de Lunel. Gérontologie et Société.

[14] Gouvernement français (1996). Ordonnance n°96-346 du 24 avril 1996 portant réforme de l'hospitalisation publique et privée.

[15] Gouvernement français (1998). Loi n°98-1194 du 23 décembre 1998 de financement de la sécurité sociale pour 1999.

[16] Gouvernement français (2001). Loi n°2001-1246 du 21 décembre 2001 de financement de la sécurité sociale pour 2002.

[17] Daniel C, Delpal B, Lannelongue C (2006). Contrôle et évaluation du fonds d'aide à la qualité des soins de ville (FAQSV) et de la dotation de développement des réseaux (DDRrapport définitif.

[18] Présidence de la République (2008). Plan «Alzheimer et maladies apparentées» 2008–2012.

[19] Challis D, Darton R, Hughes J, Stewart K, Weiner K (2001). Intensive care-management at home: an alternative to institutional care?. Age Ageing.

[20] Bras P, Duhamel G, Grass E (2006). Améliorer la prise en charge des malades chroniques: les enseignements des expériences étrangères de «disease management».

[21] Vedel I, De Stampa M, Bergman H, Ankri J, Cassou B, Mauriat C (2009). A novel model of integrated care for the elderly: COPA, Coordination of Professional Care for the Elderly. Aging Clinical and Experimental Research.

[22] Somme D, Trouve H, Couturier Y, Carrier S, Gagnon D, Lavallart B (2008). Prisma France: programme d'implantation d'une innovation dans un système de soins et de services aux personnes en perte d'autonomie. Adaptation d'un modèle d'intégration base sur la gestion de cas. Revue d'Epidemiologie et de Sante Publique.

[23] Hebert R, Raiche M, Dubois MF, Gueye NR, Dubuc N, Tousignant M (2010). Impact of PRISMA, a coordination-type integrated service delivery system for frail older people in Quebec (Canadaa quasi-experimental study. The Journals of Gerontology series B Psychological Sciences and Social Sciences.

[24] Hebert R, Durand PJ, Dubuc N, Tourigny A (2003). PRISMA: a new model of integrated service delivery for the frail older people in Canada. The International Journal for Integrated Care [serial online].

[25] Challis D, Brodsky J, Habib J, Hirschfeld M (2003). Achieving co-ordinated and integrated care among long term care services: the role of care management.

[26] Somme D, Balard F, Couturier Y, Gagnon D, Saint Jean O, Trouvé H (2011). Prisma France: projet pilote d'intégration et de gestion de cas.

